# Calcium-activated nucleotides 1 (CANT1)-driven nuclear factor-k-gene binding (NF-ĸB) signaling pathway facilitates the lung cancer progression

**DOI:** 10.1080/21655979.2021.2003131

**Published:** 2022-01-22

**Authors:** Fangfang Gao, Xiufeng Hu, Wenjing Liu, Hongbo Wu, Yu Mu, Yanqiu Zhao

**Affiliations:** Department of Internal Medicine, Henan Cancer Hospital, Affiliated Cancer Hospital of Zhengzhou University, Zhenzhou, China

**Keywords:** CANT1, proliferation, colony formation, NF-ĸB, lung cancer

## Abstract

Dysregulation of calcium-activated nucleotides 1 (CANT1) has been observed in different organs. Thus, its biological function in cancer has increasingly attracted researchers. The current work aims to study the CANT1 role in lung cancer and understand the underlying pathological mechanisms. High amplification of CANT1 was observed in lung adenocarcinoma (LUAD) and lung squamous cell carcinoma (LUSC) tissues compared to normal tissues. The high-CANT1 patients showed a dismal prognosis in comparison with the low-CANT1 patients. Highly expressed CANT1 was significantly associated with the N stage of LUSC patients. Ectopic expression of CANT1 conspicuously increased the proliferation and viability of A549 cells. Conversely, CANT1 depletion resulted in adverse effects in H1299 cells. CANT1 depletion also resulted in the retardation of tumor growth in vivo. Mechanically, we found that CANT1 could elevate NF-ĸB (nuclear factor-k-gene binding) transcriptional activity in a concentration-dependent manner. This regulatory relationship was also established by the Western blot technique. Inhibiting NF-ĸB can significantly blunt the increased NF-κ-B Inhibitor-α (IκBα) expression caused by CANT1 overexpression in A549 cells. In conclusion, highly amplified CANT1 promotes the proliferation and viability of lung cancer cells. We also elucidate a new signaling axis of CANT1-NF-ĸB in lung cancer. This approach might be a promising strategy for lung cancer treatment.

## Graphical abstract

**Figure:**
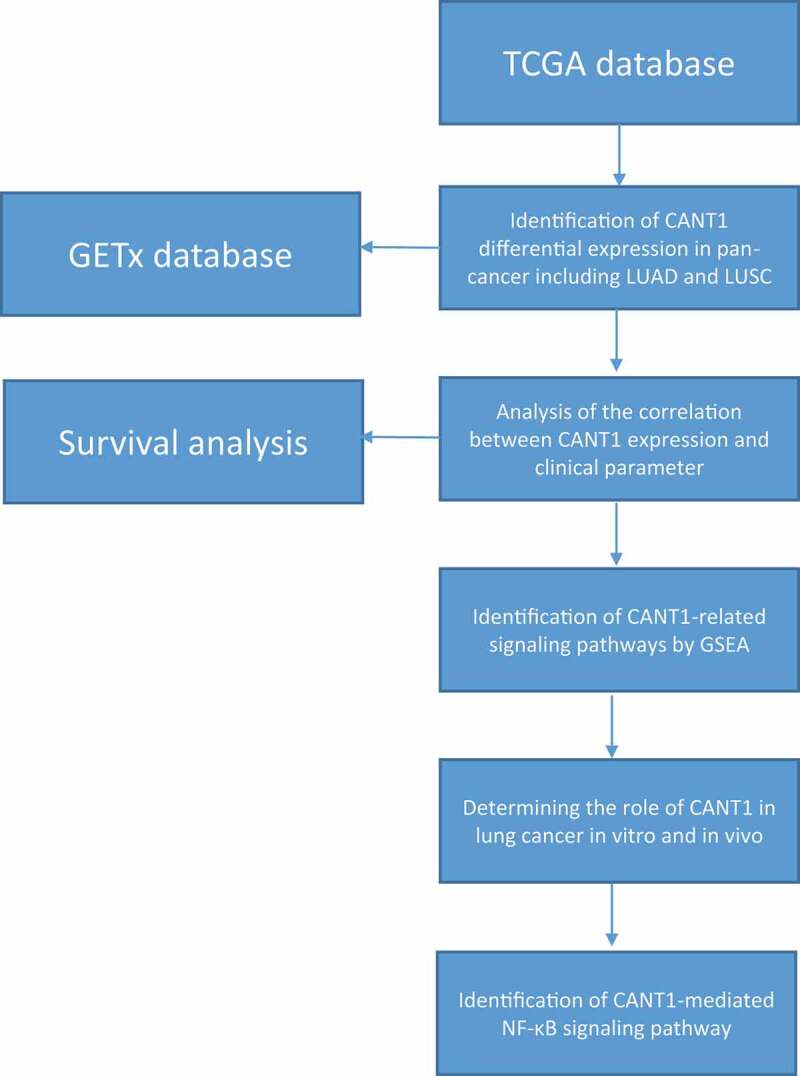

## Introduction

Lung cancer (LC) is the first most commonly diagnosed malignancy that originates from the respiratory system [1,2]. According to the latest cancer statistics, in 2020, in the United States, the estimated incidence and mortality due to LC are 228,820 and 35,720, respectively [1]. Approximately 85% of LCs are non-small cell lung cancer (NSCLC) with a 5-year survival rate of 15% [3]. Though mortality has dropped in the US, developing countries such as China in recent years have recorded higher mortalities [4]. Both environmental exposures and genetic factors increase the global burden of cancers [5]. Therefore, untangling the molecular mechanisms underlying the pathology of LC is urgently required.

Calcium-activated nucleotidase 1 (CANT1) is a calcium-dependent enzyme that shares high sequence homology with the apyrase gene. Slightly different from apyrase, CANT1 presents a preference for uridine diphosphate (UDP), not adenosine diphosphate (ADP). CANT1 mutations lead to UDP accretion with consequent suppression of glycosyltransferase reactions. Due to its role in Ca^2+^ homeostasis, it is also acknowledged as a critical modulator of the endoplasmic reticulum-Golgi apparatus and influences protein folding and degradation processes [6]. A pile of evidence has associated CANT1 mutants or variants with several human genetic disorders such as skeletal dysplasia [7], desbuquois dysplasia [6], and cancers [8]. CANT1 overexpression was detected in renal cell carcinoma [9]. Furthermore, CANT1 might be a promising target for interventions of patients with TP53-mutant hepatocellular carcinoma [10]. One gene set analysis showed that CANT1 played a role in purine and pyrimidine antimetabolites in cancer treatment [11]. All these findings suggest that CANT1 plays a role in cancer progression.

Nuclear factor-k-gene binding (NF-ĸB) is one of the documented signaling transduction cascades ‘hijacked’ to drive tumor cell survival and their oncogenic growth [12]. Once activated, its responsive coactivators release NF-ĸB transcriptional factor, accelerate its nuclear translocation, and impact the expression of branched intracellular effectors related to cell-biological properties. In lung cancer, hyperactivation of NF-ĸB was frequently observed as well as regarded as an indicator of an advanced tumor stage and unpromising survival outcome in patients [13]. It is noteworthy that NF-ĸB activation is triggered by diverse extracellular stimuli, including endoplasmic reticulum (ER) stress in lung cancer [14]. Cali et al. (2012) defined CANT1 as an ER-Golgi body resident Ca^2+^-dependent enzyme implicated in protein glycosylation [15]. Ahmmed et al. (2019) highlighted the role of the AKT/NF-ĸB signaling pathway in aberrant glycosylation of lung cancer cells [16]. These findings suggest that the interplay between CANT1 and NF-ĸB pathway plays a role in lung carcinogenesis. Therefore, we hypothesized that CANT1 drive lung cancer progression by the NF-ĸB signaling pathway. To address this assumption, we first analyzed CANT1 expression status and prognostic association with lung cancer individuals by a series biotinformatics analysis. Furthermore,we also investigated the effect of CANT1 overexpression and knockout on *in vitro* proliferation of A549 and H1299 human cancer cell lines and in vivo effect. Additionally, the effect of CANT1 to NF-ĸB signaling pathway involved in lung cancer cells was also ascertained.

## Methods

### Antibodies and plasmids

Mouse monoclonal anti- FLAG antibody, anti-IκBα antibody, and anti-GAPDH antibody were obtained from Proteintech Group, Inc, Wuhan, China. Rabbit anti-CANT1 antibody was obtained from Abcam Company (USA). Horseradish peroxidase (HRP)-conjugated secondary antibodies were purchased from Invitrogen, Beijing, China. NF-ĸB reporter luciferase plasmid (REPOTMNF-κB) genes were purchased from Yeasen Biotechnology Co., Ltd. Shanghai, China.

### Cell culture and transfection

H1299 human NSCLC cells, A549 human lung cancer, and human embryonic kidney cell HEK293T were purchased from the Wuhan Cell Bank of Wuhan University (Wuhan, China). All the cells were incubated in Dulbecco’s Modified Eagle’s Medium (DMEM), added 1% penicillin-streptomycin solution and 10% fetal bovine serum. The culture condition was 37°C and a 5% CO_2_ humidified atmosphere. The calcium phosphate transfection kits were used for transfection as described in the product specification.

### CANT1 genetic depletion in H1299 cells

CANT1 genetic depletion was achieved with the aid of CRISPR/Cas9 genome editing technology. A single guide RNA (sgRNA) targeted CANT1 exon 1:AAGGACGAGCGTCTGTACGT was designed via https://portals.broadinstitute.org. The sgRNA oligo and its nontargeted scramble control were synthesized at Tianyi Huiyuan Biotech Co, Ltd, Wuhan, China, and were ligated into lentiCRISPRv2. The resulting product was transfected 50–60% HEK293T cells with the packaging plasmids. At 48 h post-transfection, the lentiviruses were obtained and infected to 50% H1299 cells. After 10-day puromycin screening, the homogenous pool of CANT1 knockout cells was incubated in a 10-cm Petri dish, and single clones were selected. Immune bolt experiments were performed to confirm CANT1 knockout.

### CANT1 overexpression in A549 cells

CANT1 encoding sequence was isolated from mRNA of A549 cells by reverse transcription and ligated into a pCMV-Tag2 expression plasmid. The recombinant vectors for expressing FLAG-tagged CANT1 were transfected into subconfluent A549 cells for 48 h. The transfected cells were subjected to kanamycin selection (50 mg/L) for seven days. Finally, immunoblot experiments and Sanger sequencing were performed to validate the CANT1 overexpression.

### Immunoblot analysis

The cell protein sample was prepared by sodium dodecyl sulfate (SDS) sample buffer and quantitatively evaluated by the pierce protein quantitation assay kit. Equivalent samples (20 µg) were electrophoresed to 12% SDS-PAGE and electrotransferred to a polyvinylidene fluoride (PVDF) membrane. Before incubation with anti-FLAG, anti-CANT1, or anti-GAPDH for 24 h, the membrane was subjected to blockage with 5% skimmed milk. Subsequently, the blot was detected using secondary antibody-conjugated horseradish, and immunoreactive bands were captured using ECL and X-ray films.

### Cell proliferation assay

The CCK8 kit was employed to assess the proliferative capacity of lung cancer cells as per the manufacturer’s protocol. 10 µL of cell suspension containing 1 × 10^3^ cells were seeded in each well of 96-well plates. On days 1, 3, and 5 post culture, 10 µL of CCK8 solution was added to different wells and continuously cultured for another 1 h. The optical density at 450 nm was recorded using an enzyme-labeling instrument (Promega).

### Colony formation assay

Analysis of cell viability of lung cancer cells was performed by colony formation assays. A total of 2 × 10^3^ cells/well were seeded on the six-well plates and maintained for 14 days. The cells were incubated with 4% methyl alcohol for 10 min before staining with 5% crystal violet solution. Cell clones >50 were recorded.

### Dual-Luciferase reporter assay

A Dual-Luciferase reporter assay system was employed to quantify NF-ĸB -driven transcriptional activity in A549 cells and H1299 cells. First, 1 × 10^3^ cells were cultured in 12-well plates and cotransfected with 10ng REPOTMNF-κB, plasmid pRenilla, and gene CANT1-overexpressing constructs (0, 100, 200, 400ng). At 48 h post-transfection, luciferase was examined by dual-luciferase kits (Promega, Beijing, China) as directed by the manufacturer. NF-ĸB -driven luciferase was normalized against pRenilla.NF-ĸB-driven transcriptional activity was also detected in CANT1-deficient H1299 cells, as described above.

### In vivo tumorigenesis model

Twelve 5-week-old nude male mice (weight, about 20 g) were supplied from the Experimental Animal Center of Wuhan University (Wuhan, China). The mice were caged individually at 18–23°C with 12-h light and dark cycles, and 60% humidity and had free access to water and food. H1299 cells or KO-1 cells (8 × 10^6^) were administered into the subcutaneous tissues of the right flank of subjects. After 28 days, the mice were euthanized. Tumors were aseptically taken out and weighed. The care for animals was in accordance with institutional guidelines and was approved by the Ethics Committee of Affiliated Cancer Hospital of Zhengzhou University, China.

### Bioinformatics analyses

All bioinformatics analyses were performed using R (R version 3.6.3) and visualized using ggplot2 with p ≤ 0.05 indicating statistical significance. The CANT1 expression profile in pan-cancer was analyzed based on pan-cancer patient data acquired from TCGA and GTEx databases. Meanwhile, to verify CANT1 expression in lung cancer patients, we employed the TCGA and GTEx databases to compare the CANT1 mRNA expression in lung adenocarcinoma (LUAD) tissues and lung squamous cell carcinoma (LUSC) with their paired or unpaired normal paracancerous tissues. To determine the association of CANT1 expression with the prognosis of lung cancer patients, the patients for the TCGA-LUSC cohort or TCGA-LUSC cohort were categorized into CANT1-high and CANT1-low groups. Survival prediction was plotted based on the clinical information from LUSC patients and LUAD patients.

Gene set enrichment analysis (GSEA)

The LUAD patients from TCGA cohorts were closed for GSEA. The subjects were grouped into the high- and low-CANT1 groups according to its medium expression. The clusterProfiler R package was used to analyze the differential expression genes between two groups. Gene sets with P < 0.01 and false discovery rate<0.1 were considered significant.

### Statistical analysis

All data are expressed in the mean ±SD and are analyzed with PRISM version 9.0. An unpaired or paired t-test was adopted to compare the difference between the two groups. One-way ANOVA analysis with Bonferroni posttest was used to evaluate the statistical difference between multiple groups. To balance statistical error rates, Bonferroni corrections were used for all pairwise comparisons. A p-value < 0.05 was considered to be of statistical significance.

## Results

### Upregulation of CANT1 in LUAD and LUSC

**Figure 1.**
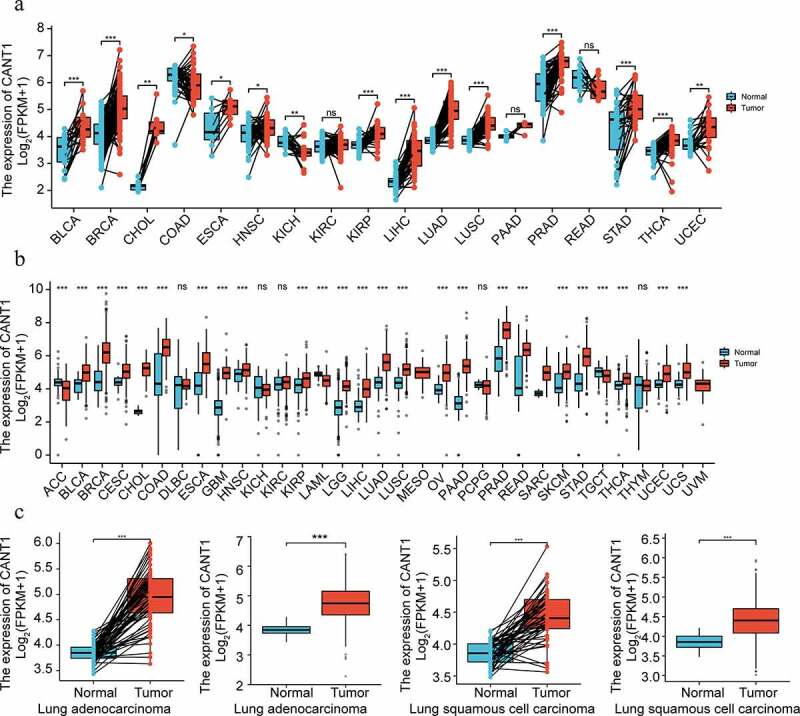
CANT1 is increased in LC. (a). CANT1 was frequently highly expressed in most cancers in TCGA and GTEX database by bioinformatics analysis. (b). CANT1 was frequently highly expressed in most cancers in TCGA database by bioinformatics analysis. (c). The expression level of CANT1 was detected in TCGA-LUAD cohort. (d). The expression level of SREBP1 was detected in TCGA-LUAD compared with normal TCGA and GTEx clinical samples. (e). The expression level of CANT1 was detected in TCGA-LUSC cohort. (f). The expression level of CANT1 was detected in TCGA-LUSC compared with normal TCGA and GTEx normal clinical samples.

**Figure 2.**
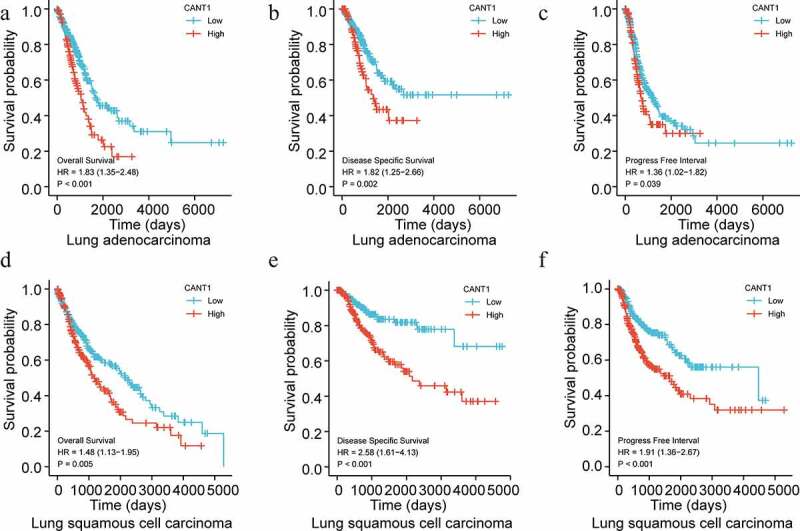
Survival plots for CANT1 in LC. Overall survival (a), Progression-free survival (b) and disease specific survival (c) evaluated by Kaplan–Meier curves on LUAD patients from the TCGA dataset. Overall survival (d), Progression-free survival (e and d) disease specific survival (f) evaluated by Kaplan–Meier curves on LUSC patients from the TCGA dataset.

Initially, we disclosed the CANT1 expression according to TCGA Pan-Cancer dataset. As shown in Figure 1a, CANT1 was highly expressed in most solid cancer tissues compared with that in the paracancerous tissues. Based on TCGA and GTEx database, CANT1 was highly expressed in most solid cancer tissues compared with the normal tissues (Figure 1b). Both results suggested that CANT1 might play an important role in cancer malignancy. In line with the results from the pan-cancer analysis, we confirmed that CANT1 was amplified in LUAD and LUSC tissues compared with the paracancerous tissues or normal tissues from TCGA or GTEx (Figure 1c and d). Kaplan-Meier survival analysis demonstrated that high-CANT1 LUAD or LUSD patients have a lower probability of overall survival, disease-specific survival, and progress-free interval survival (Figure 2). Highly expressed CANT1 was tightly associated with poor N stage and M stage in the LUSA patients (Table 1). These findings suggest the role of CANT1 in oncogenicity in lung cells.

**Table 1.** Logistic analysis of the association between CANT1 expression and clinical characteristics of Lung squamous cell carcinoma (LUSC)

Characteristic	Low expression of CANT1	High expression of CANT1	p
n	251	251	
T stage, n (%)			0.870
T1	59 (11.8%)	55 (11%)	
T2	145 (28.9%)	149 (29.7%)	
T3	37 (7.4%)	34 (6.8%)	
T4	10 (2%)	13 (2.6%)	
N stage, n (%)			0.006
N0	173 (34.9%)	147 (29.6%)	
N1	57 (11.5%)	74 (14.9%)	
N2	15 (3%)	25 (5%)	
N3	0 (0%)	5 (1%)	
M stage, n (%)			0.065
M0	201 (48%)	211 (50.4%)	
M1	6 (1.4%)	1 (0.2%)	
Age, median (IQR)	68 (62, 74)	68 (62, 73)	0.415

RNA-seq data of CANT1 gene expression in Lung squamous cell carcinoma (LUSC) from TCGA (https://tcga-data.nci.nih.gov/tcga/).

### *GSEA identifies a* CANT1-*related signaling pathway*

**Figure 3.**
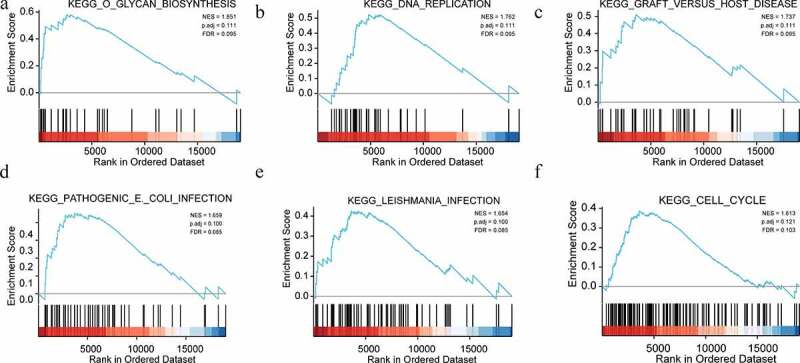
Enrichment plots from GSEA. (a). Glycan biosynthesis; (b). DNA replication; (c). Graft versus host disease; (d). Pathogenic (e). coli infection; (E). Leishmania infection; (f). cell cycle.

To further reveal the function of CANT1 in lung cancer, GSEA analysis was conducted based on TCGA- LUSC and TCGA-LUAD databases. In CANT1-high LC patients, GO terms enriched included Glycan biosynthesis, DNA replication, Graft versus host disease, pathogenic *E. coli* infection, *Leishmania* infection, and cell cycle (Figure 3). These findings suggested that highly expressed CANT1 might be associated with the key processes which favor carcinogenesis and progression.

### *Overexpression of CANT1 promotes* in vitro *proliferation and colony formation in A549 cells*

**Figure 4.**
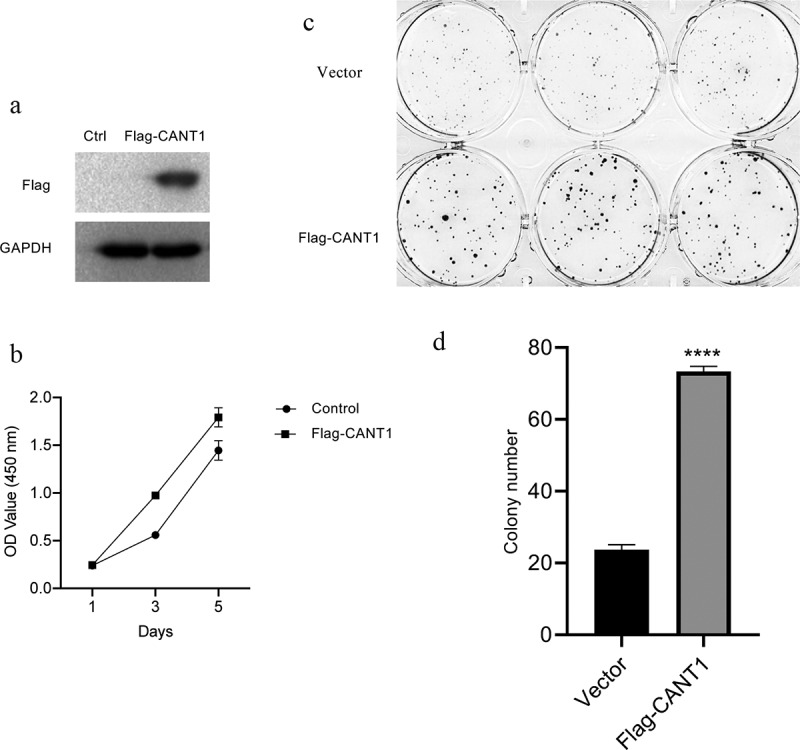
Overexpressing CANT1 inhibits LC cell proliferation and colony formation in vitro. (a).CANT1 enforced expression was confirmed by western blot. (b). CCK8 forming assays verifying the proliferation of A549 cells transfected with CANT1-overexpressing vector or the scrambled vector, and (d). Number of colonies A549 cells transfected with CANT1-overexpressing vector or the scrambled vector.A549 cells transfected with CANT1-overexpressing vector or the scrambled vector.

To determine the functional role of CANT1, we established A549 cells with FLAG-tagged CANT1 stably overexpressed (Figure 4a). CCK8 assays demonstrated that highly expressed CANT1 could substantially increase the proliferative capacity compared with A549 cells transfected with the scrambled control (Figure 4b). Colony formation assays demonstrated significantly fewer colonies in CANT1 overexpressing A549 cells in comparison with those in A549 transfected with the scrambled control (Figure 4c and d).

### CANT1 deficiency reduces H1299 cell proliferation and colony formation in vitro and in vivo

**Figure 5.**
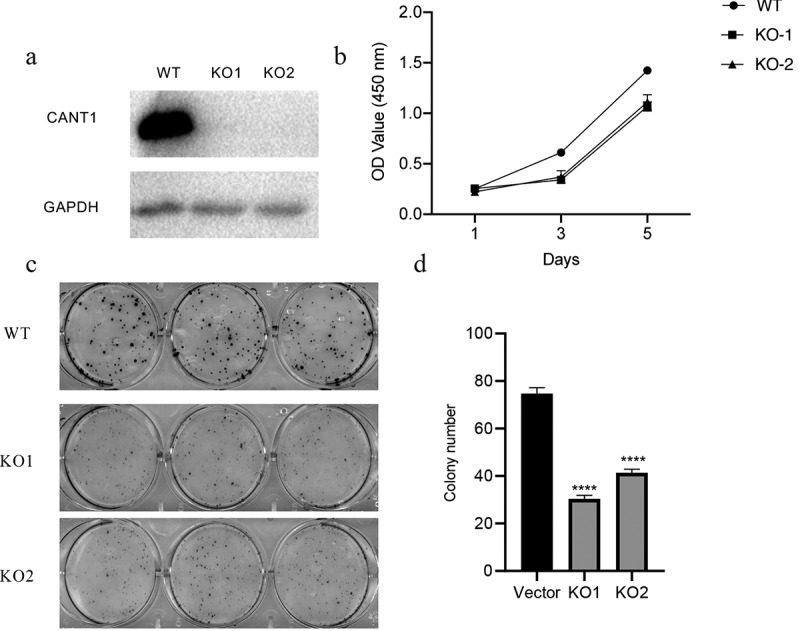
CANT1 deficiency impairs *in vitro* proliferation and colony formation. (a). the CANT1 expression in CANT1-knockout H1299 cells (KO-1 and KO-2) and the control cells. (b). The proliferative rate of H1299 cells was analyzed by CCK8 assays after CANT1 deficiency. (c and d). The colony-forming ability of H1299 cells was analyzed by colony-forming assay after CANT1 deficiency.

**Figure 6.**
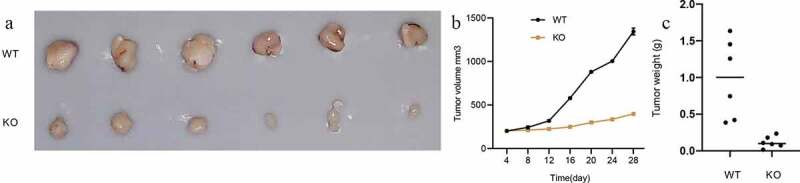
CANT1 depletion decreases tumorigenesis in vivo. (a). Images of the isolated tumors from mice of the vector and CANT1-depletion groups. (b). The tumor volume in mice was plotted every 4 days. (c). Tumor weights of the primary tumor xenografts upon euthanasia at day 28.

The CRISPR-Cas9 mediated methods of gene editing were utilized to deplete the CANT1 expression in H1299 cells. Following transfection, immunoblot analyses confirmed knockout efficiency (Figure 5a). CCK8 assay was then performed to evaluate the effect of CANT1 depletion on the H1299 cell proliferative behaviors (Figure 5b). The findings showed that CANT1 deficiency restrained the proliferative rate of H1299 cells. Colony formation assay similarly showed that CANT1 deficiency weakened the H1299 cells colony-forming ability (Figure 5c, d). Overall, our findings indicated the role of CANT1 in lung cancer development. To further elucidate the role of CANT1 in lung cancer, we established a xenograft model bearing CANT1-deficient H1299 cells or normal H1299 cells. The results demonstrated that CANT1 depletion inhibited tumor growth, resulting in reduced tumor size and tumor weight (Figure 6). Taken together, CANT1 functioned as an oncogene in lung cancer tumorigenesis and progression.

### CANT1 activates NF-ĸB signaling in LC cells

**Figure 7.**
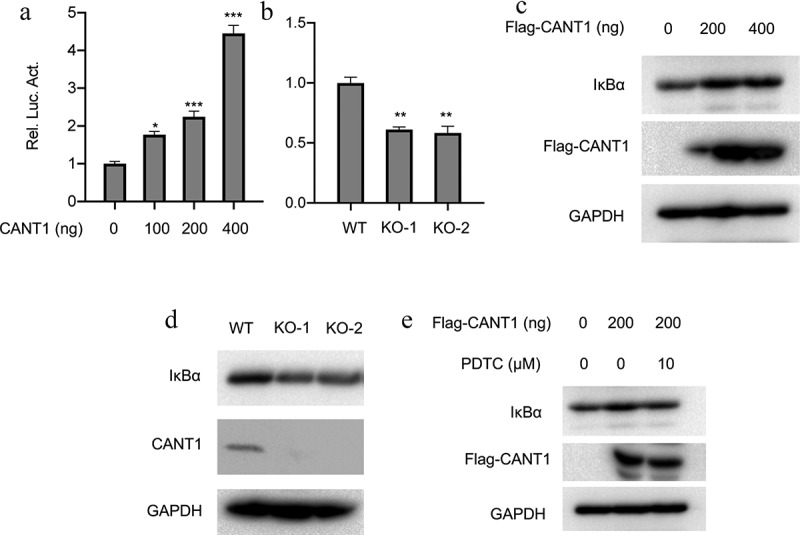
CANT1 activates the NF-ĸB signaling pathway in lung cancer cells. (a). The NF-ĸB activity in A549 cells was evaluated by Luciferase reporter assays in the presence (0,100,200, and 400ng) CANT1-overexpressing plasmids. (b). The NF-ĸB activity in CANT1-deficient A549 cells was assessed by Luciferase reporter assays. (c). the IκBα expression was analyzed by immunoblot assays in A549 cells after transfection with CANT1-overexpressing plasmids (200,400ng). (d). The IκBα and CANT1 expression was examined by western blot in CANT1-deficient A549 cells. (e). The IκBα expression in A549 cells transfected with 200ng CANT1-overexpressing plasmids was assessed by Western blot post-treatment with 10 µM of an NF-ĸB inhibitor: pyrrolidine dithiocarbamate (PDTC) or not .*p < 0.05, ** P < 0.01, ***p < 0.001.

Considering the possible interaction of CANT1 and NF-ĸB in glycosylation of lung cancer cells, we further checked the effect of CANT1 on NF-ĸB signaling. Interestingly, we found that CANT1 could dose-dependently increase the NF-ĸB -mediated luciferase activity in HEK293T cells (Figure 7a). Moreover, we validated the association between CANT1 and NF-ĸB signaling using luciferase reporter assays and found a profound decrease in NF-κB reporter activity in knockout 1 (KO-1) and KO-2 cells (Figure 7b). We further determined whether there is an interplay of CANT1 and IκBα, which is a critical factor implicated in the NF-ĸB signaling pathway. As shown in Figure 7c, overexpression of CANT1 pronouncedly induced accumulation of inhibitory- kappa B–alpha protein (IκBα). Conversely, genetic depletion of CANT1 downregulated the CANT1 expression. PDTC could restrain the phosphorylation of IκBα, thereby resulting in inhibition of NF-ĸB activity (Figure 7d). To further understand the underlying mechanism, we adopted 10 µM PDTC (Pyrrolidinedithiocarbamic acid) to treated CANT1-overexpressing A549 cells and observed that PDTC treatment had offset the increased IκBα expression induced by CANT1 overexpression (Figure 7e). This suggested that the facilitatory effects of CANT1 to NF-ĸB activation were nullified by the inhibitory effect of PDTC.

## Discussion

CANT1 is a soluble UDP-preferring nucleotidase, and its dysfunction contributes to protein instability and tumor susceptibility in several cancers. However, CANT1 expression in LC tissue and its prognostic value remained poorly understood. Previously, accumulating reports have verified the prognosis by bioinformatic analysis [17–19]. In the present work, the CANT1 expression profile in pan-cancer was evaluated using the TCGA or GTEx database. Robust expression of CANT1 was observed in most solid cancers, including LUAD and LUSC. Furthermore, in 2011, Gerhardt et al. found that high-CANT1 is related to favorable prognosis in cohort of >1000 prostate cancer patients. In lung squamous carcinoma, Qiao et al. demonstrated that CANT1 is positively related with epithelial-mesenchymal transition (EMT)-associated biomolecules, which are correlated with poor prognosis of cancer patients. Based on TCGA and/or GTEx database, Qiao et al. found that CANT1 was highly expressed in LUSC patients [20]. However, the prognosis in LC remains unknown. In our present work, survival shown in Kaplan–Meier curves indicated that highly expressed CANT1 is an inferior prognosis of LUAD and LUSD patients. Furthermore, high-CANT1 is segregated with N stage and M stage in LUSD. All these data suggest its oncogenicity in LC.

Sustaining chronic proliferation of cancer cells was a typical pathological feature of LC, facilitating its tumorigenesis. To further explore the CANT1 role in LC cells, we manifested that CANT1 genetic inactivation dramatically impaired the proliferative and colony-forming capacities of H1299 cells using CRISPR/Cas9 technology. These findings confirm previous investigations that reported the inhibition of CANT1 silenced on the cellular proliferation of renal cell carcinoma and prostate cancer [9,21]. Contrary to these findings, overexpressing CANT1 in A549 cells increases cell proliferation and colony formation *in vitro*. This gave a clue to support previous findings that demonstrate an association of CANT1 with mutant tumor protein p53(TP53) gene that plays a putative role in cellular proliferation in hepatocellular carcinoma [10]. The in vitro results were further verified in vivo. These findings indicate that CANT1 is a crucial modulator of the LC progression.

To further understand the biofunction of CNAT1 in LC, we performed the GSEA analysis based on the TCGA database. As indicated by the enrichment analysis, CANT1 was functionally related to glycan biosynthesis, DNA replication, graft versus host disease, pathogenic *E. coli* infection, *Leishmania* infection, and cell cycle. Accumulating evidence has demonstrated that the disruption of glycan biosynthesis and DNA replication are beneficial to the malignant behaviors of cancer cells, such as cell cycle, migration, proliferation [22,23]. Furthermore, previous studies demonstrated that *Leishmania* infection and pathogenic *E. coli* infection are two kinds of pulmonary infections of NSCLC patients [24,25]. During this inflammatory response, increasing pleiotropic pro-cancer cytokines, such as IL-33, IL-6 are secreted and act pro-tumor cytokine to facilitate LC progression. The activation of NF-ĸB signaling events is frequently observed in cancer-related inflammation. Furthermore, the function of the NF-ĸB signaling pathway in malignant transformation and cancer progression has been examined in pan-cancers, including LC. Considering the involvement of NF-ĸB and CANT1 in ER stress [14,26], we clarified whether the overexpression or depletion of CANT1 influences the NF-ĸB transcriptional activity by luciferase reporter assays. Our investigation evidenced that the overexpression of CANT1 could dose-dependently increase the NF-ĸB-meditated luciferase activity in A549 cells as well as the expression of IκBα. Conversely, we uncovered that the genetic inactivation of CANT1 in H1299 cells clearly suppressed NF-ĸB transcriptional activity, as well as the NF-ĸB protein expression. Furthermore, we observed a counteracting effect of PDTC on the increased IκBα expression in A459 cells transfected by CANT1 overexpressing vectors. Thus, we suggest that CANT1 is a positive regulator of the NF-ĸB signaling pathway, and its dysregulation causes LC progression. To our knowledge, our investigation firstly revealed the underlying mechanism of CANT1 in cancer cells, although earlier investigation showed CANT1 role in tumor cell malignant behaviors.

## Limitation

There are a few limitations of the present study. Firstly, validation using clinical samples is needed. Secondly, in vivo experiments are needed to test the alternation of the NF-ĸB signaling pathway. Additionally, the regulatory mechanism of CANT1 in lung cancer is complex; further work will focus on it.

## Conclusion

Conclusively, our investigations sustain the notion that CANT1 is a tumor-promoting oncogene whose dysfunction impacts the LC cell proliferation and colony formation. The current study highlights a novel mechanistic insight for LC partially controlled by CANT1/NF-ĸB axis and presents the novel CANT1 targeted therapies for LC treatment.

## Data Availability

The data that support the findings will be available in this manuscript and TCGA and GTEx database. https://www.cancer.gov/about-nci/organization/ccg/research/structural-genomics/tcga
